# Ischemic Stroke in an HIV Positive Patient: An Initial Presentation of Neurosyphilis

**DOI:** 10.1155/2018/2410154

**Published:** 2018-02-13

**Authors:** Kalimullah Jan, Rebecca Hoe Hui Min, Tan Seow Yen, Shekhawat Ravindra Singh

**Affiliations:** ^1^Department of Medicine, Changi General Hospital, Singapore; ^2^Department of Infectious Diseases, Changi General Hospital, Singapore; ^3^Department of Neurology, Changi General Hospital, Singapore; ^4^National Neuroscience Institute, Singapore

## Abstract

Ischemic stroke occurring in patients with human immunodeficiency virus (HIV) needs to be approached with a vast differential diagnosis in mind. We report a case of middle-aged male patient with immune reconstituted HIV on therapy without known cardiovascular risk factors who had a right middle cerebral artery territory infarct. After a thorough evaluation, he received a final diagnosis of neurosyphilis-associated vasculitis leading to stroke. He recovered without any neurological deficits following treatment with intravenous benzylpenicillin. Neurosyphilis is an easily diagnosed and treatable cause of a stroke that can be an initial presentation of neurosyphilis but requires a high index of suspicion.

## 1. Case Report

A 57-year-old Chinese male presented to the Emergency Department (ED) of a tertiary hospital with complaints of transient left upper and lower limb weakness associated with a left facial droop and slurring of speech that had lasted for half an hour. He denied any history of similar events. He had a known history of human immunodeficiency virus (HIV) infection on highly active antiretroviral therapy (HAART) for the past four years. He was a nonsmoker. There was no family history of stroke.

On initial presentation, he was hemodynamically stable with blood pressure of 165/98 mmHg. Physical examination revealed dysarthria with a left facial droop and left-sided pronator drift. The rest of the examination was unremarkable. His capillary blood glucose was normal. His National Institutes of Health Stroke Scale (NIHSS) score on arrival to ED was 3 points.

Urgent noncontrast computed tomography (CT) of the brain was obtained in ED within minutes of arrival. This showed acute right middle cerebral artery (MCA) territorial infarct without haemorrhagic conversion. There was presence of mild right frontoparietal mass effect. His ASPECTS (Alberta Stroke Programme Early CT Score) was 5. In view of rapidly resolving symptoms and low NIHSS score, thrombolysis with recombinant Tissue Plasminogen Activator was not offered.

He was admitted to the acute stroke unit after starting aspirin (100 mg) and Atorvastatin (40 mg). For his HIV, he was being prescribed daily doses of abacavir, efavirenz, and lamivudine, to which he claimed compliance. His last CD4 count was 533 cells/uL (lab range: 280–1430) one year ago. The most recent HIV viral load performed a week before admission was detectable but less than 20 copies/mL.

His initial investigations revealed a normal electrocardiogram, troponin, random glucose, urea, electrolytes, liver function test, coagulation profile, and full blood count. Fasting low density lipoprotein cholesterol (LDL-c) was 2.28 mmol and his HbA1c was less than 4.3%. His subsequent inpatient blood pressure readings were normal and antihypertensive agents were not required. His chest radiograph, transthoracic echocardiography, Doppler ultrasound of bilateral carotids, and 24-hour Holter monitoring were normal.

**Figure 1:**
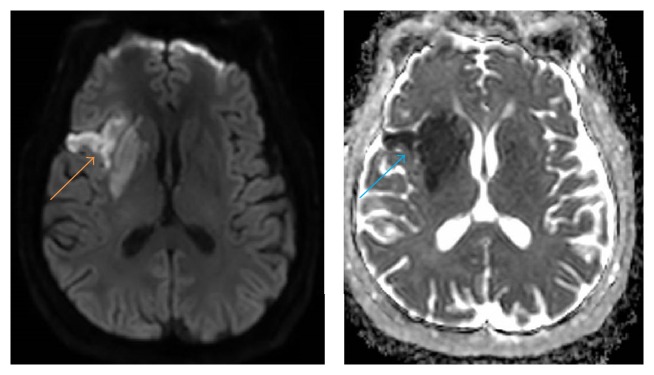
MRI brain reveals an area of diffusion restriction over the right MCA territory (orange arrow) with corresponding signal loss on Apparent Diffusion Coefficient (ADC) mapping (blue arrow), suggesting acute ischemic infarct.

Magnetic resonance imaging (MRI) of the brain was performed while he was an inpatient, revealing a right MCA territory infarct (Figure 1). Time of flight magnetic resonance angiography (ToF-MRA) showed suggestion of mild stenosis of the middistal right M1 segment of the MCA with no flow limitation.

In view of the absence of known cardiovascular risk factors, a thrombophilia and autoimmune workup was performed as shown in Table 1.

**Figure 2:**
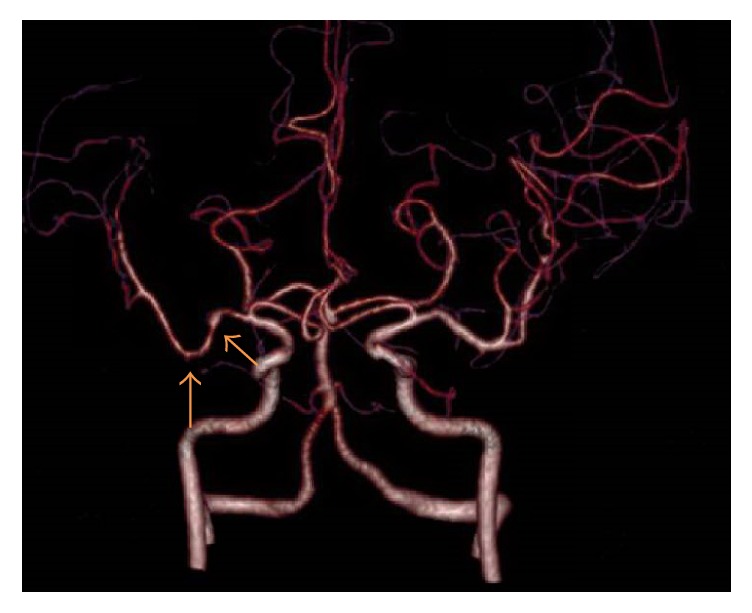
CT angiogram of the circle of Willis showing multiple areas of luminal irregularity.

CT angiography of the circle of Willis was performed to look for evidence of vasculitic phenomena. As shown in Figure 2, there was bilateral moderate luminal irregularity in the M2 branches of the MCAs (more pronounced on the right, as shown by arrows) with less pronounced changes in the A2 segments of the anterior cerebral arteries and their branch vessels, raising suspicion for small to medium vessel vasculitis.

A lumbar puncture was performed, which showed 203 white blood cells (WBC) with lymphocytes accounting for 90%. His cerebrospinal fluid (CSF) protein was raised at 0.81 g/L (0.10–0.40 g/L). CSF VDRL was positive at titres of 1 : 8 and his CSF Syphilis LIA-IgG was positive as well, thus confirming neurosyphilis.

He received a diagnosis of ischemic stroke secondary to neurosyphilitic vasculitis. The infectious diseases team was consulted and he was started on intravenous benzylpenicillin 4 million units 4 hourly for a total of 14 days. His aspirin and atorvastatin were discontinued. He recovered without any neurological sequelae during the course of his admission.

## 2. Discussion

Cerebrovascular disease is common in patients with HIV, occurring in up to 1.9% of HIV-infected patients [1]. In particular, patients with the acquired immunodeficiency syndrome (AIDS) have been reported to be at increased risk of both ischemic and haemorrhagic strokes, of which ischemic stroke is more frequent [2]. The pathogenesis of ischemic stroke in HIV is not well understood, with multiple postulated mechanisms including cardioembolism, coagulopathy, opportunistic infections, and HAART-related accelerated atherosclerosis, as well as primary vasculitis of the cerebral nervous system [3–5].

Patients with HIV are also susceptible to the atherosclerotic mechanisms affecting the majority of patients with stroke, and evaluation of stroke in HIV patients should similarly include Doppler ultrasound of the carotid vessels and cardiac evaluation. These investigations are useful to identify other conditions associated with HIV that predispose to stroke, including accelerated atherosclerosis, endocarditis, or cardiovascular complications of HIV infection [6].

MRA performed with MRI of the brain may reveal evidence of vasculitis in patients with HIV. Vasculitis in HIV is generally secondary to opportunistic infections or lymphoproliferative diseases [7]. In patients in whom the above have been excluded, HIV-related vasculitis is one consideration. This has been reported to occur in up to 20% of patients with HIV [8]. While the pathophysiology behind HIV-related vasculitis is not well defined, this condition is more commonly described in patients with low CD4 counts. HIV-related vasculitis can involve both intra- and extracranial arteries. HIV-associated intracranial vasculitis has been described as fusiform aneurysms involving the major arteries of the circle of Willis and the second- and third-order branches [9]. Tipping et al. described an association between intracranial vasculitis and low CD4 counts, while extracranial vasculitis appeared to be more common in patients with preserved CD4 counts [8]. This could reflect underlying differences in their pathogenesis.

As sexually transmitted diseases, syphilis and HIV frequently coexist. Amongst patients with HIV, those with lower CD4 counts are more likely to have concomitant neurosyphilis [10]. Central nervous system involvement is an uncommon manifestation of syphilis, occurring in less than 10% of patients with syphilis [11]. A study of HIV-infected patients by Ghanem et al. identified CD4 count of less than 350 cells/ml and lack of HAART treatment as predictors of neurosyphilis [12]. Lower CD4 counts have also been found to be associated with an increased risk of ischemic stroke [4, 7, 13]. Neurosyphilis can manifest as meningovascular syphilis, including meningitis or vasculitis, or parenchymal involvement in the form of general paresis or tabes dorsalis [14]. Meningovascular syphilis refers to the occurrence of an acute focal neurological deficit in a vascular territory of the brain or spinal cord based on clinical and/or imaging findings. Meningovascular syphilis has been reported to account for 24–53% of neurosyphilis, with a reduction in the proportion of late stages of neurosyphilis in the antibiotic era [15]. The mechanisms described include small vessel arteritis, also known as Nissl-Alzheimer arteritis, medium to large vessel arteritis, also known as Heubner arteritis, and less commonly extrinsic compression from aortic aneurysms [16, 17]. Angiographically, meningovascular syphilis tends to display focal segmental narrowing and dilatation, resulting in the appearance of “beading” [18]. However, these findings are nonspecific and can resemble findings in other medium and large vessel vasculitides, and an underlying HIV-associated vasculopathy cannot be excluded.

Serologic screening for syphilis should be performed in patients with cryptogenic stroke, particularly in those with risk factors for syphilis [14, 19]. The presence of a positive treponemal test should prompt further evaluation for neurosyphilis in the form of a lumbar puncture [20]. CSF features suggestive of neurosyphilis include lymphocytic pleocytosis, low glucose, and raised protein [14], which may occur in HIV alone as well [21]. Positive CSF VDRL is diagnostic of neurosyphilis, although the absence of CSF VDRL does not exclude it [22]. In patients with high suspicion for neurosyphilis, further evaluation in the form of CSF FTA-ABS is recommended [22].

Treatment of neurosyphilis is the same in HIV positive and negative patients and involves a 10- to 14-day course of high-dose intravenous benzylpenicillin [22]. Repeat CSF evaluation every 6 months is recommended in patients whose pretreatment CSF showed pleocytosis until the CSF cell count normalizes. Declining nontreponemal titres may be predictive of normalization of CSF white cell count [23]. Neurological deficits frequently but not always resolve following treatment. Conventional therapy including antiplatelets and statins for secondary prevention of stroke has not been well studied in patients with HIV or neurosyphilis. However, it is reasonable to suggest that such therapy be implemented in the absence of contraindications, bearing in mind the potential for drug interactions with HAART [6, 24, 25]. With the increasing life expectancy of patients with HIV, the incidence of stroke in patients with HIV may be expected to rise. As such, there may be a role for further investigation into the management of stroke in patients with HIV.

In our patient, despite his age, the absence of cardiovascular risk factors as well as his known diagnosis of retroviral disease prompted further evaluation which included not just screening for syphilis but also for other prothrombotic states. His positive serum VDRL and TPPA serologies in the absence of clinical features of syphilis confirmed the diagnosis of latent syphilis. Further history from the patient was unrevealing for any recent sexual encounters or positive contact history. His subsequent positive CSF VDRL confirms the diagnosis of neurosyphilis, while his cerebrovascular imaging was consistent with that of vasculitis, leading to the diagnosis of ischemic stroke secondary to meningovascular syphilis. As this patient had no other cardiovascular risk factors, and his LDL-c was normal, the decision was made to discontinue his aspirin and statin. Our patient's HIV infection was well suppressed with HAART. This suggests that screening for neurosyphilis in HIV patients who present with ischemic stroke is essential regardless of the degree of immunosuppression.

## 3. Conclusion

In summary, patients with HIV are predisposed to cerebrovascular diseases through a variety of aetiologies, of which neurosyphilis is an easily diagnosed and treatable cause. While these mechanisms of stroke are commoner in patients with AIDS, in the absence of identifiable risk factors, care should be taken to exclude an underlying cause in order to ensure correct treatment and prevention of recurrent episodes of debilitating stroke. Also, it is to be noted that stroke can be an initial presentation of neurosyphilis and requires a high index of suspicion.

## Figures and Tables

**Table 1:** Serum thrombophilia and autoimmune tests.

Test	Result
Serum Venereal Disease Research Laboratory (VDRL)	Reactive, titres of 1 : 64
Serum Treponema pallidum particle agglutination (TPPA) test	Reactive
Anti-cardiolipin IgM antibody	48.93 (MPL^1^): likely false positive *(Lower normal limit for the laboratory is 12.5 MPL, indeterminate: 12.5–20)*
Anti-cardiolipin IgG antibody	<9.40 [GPL] *Negative < 15 GPL*^*2*^
Anti-thrombin III	Normal
Protein C	Normal
Protein S (functional)	Low at 60% (range: 75–130%)
Factor V Leiden mutation	Not detected
Anti-nuclear antibodies (ANA)	Positive at titres of 1 : 80
Anti-double stranded DNA	Negative
Anti-neutrophil cytoplasmic antibodies (ANCA)	Negative
*(anti-myeloperoxidase, anti-proteinase 3)*	
Extracted nuclear antigen (ENA) profile	Negative for anti-SSA^3^, anti-SSB^4^, anti-Scl70^5^, anti-ribonucleic protein, anti-Smith antigen, and anti-Jo1^6^

^1^MPL = 1 microgram/ml IgM, ^2^GPL = 1 microgram/ml IgG, ^3^anti-Sjögren's syndrome-related antigen A, ^4^anti-Sjögren syndrome type B antigen, ^5^anti-topoisomerase I, and ^6^anti-histidyl tRNA synthetase.
